# Immune System Modeling and Related Pathologies

**DOI:** 10.1155/2012/274702

**Published:** 2012-12-17

**Authors:** Francesco Pappalardo, Vladimir Brusic, Holger Fröhlich

**Affiliations:** ^1^Dipartimento di Scienze del Farmaco, Università degli Studi di Catania, Viale Andrea Doria, 6, 95125 Catania, Italy; ^2^Cancer Vaccine Center, Dana-Farber Cancer Institute, 77 Avenue Louis Pasteur, HIM 401, Boston, MA 02115, USA; ^3^University of Bonn, Bonn-Aachen International Center for IT (B-IT), Algorithmic Bioinformatics, Dahlmannstr, 2, 53113 Bonn, Germany

Revolutions in biotechnology and information technology have produced enormous amounts of biomedical data. Processing and analysis of these data are accelerating the expansion of our knowledge of biological systems. These advances are changing the way biomedical research, development, and applications are done. Clinical data complement the basic biology data, enabling detailed descriptions and modeling of various healthy and diseased states, disease progression, and the responses to therapies. The availability of data representing various biological states, processes, and their time dependencies enables the study of biological systems at various levels of organization, from molecule to whole organism, and even at population levels. Multiple sources of data support a rapidly growing body of biomedical knowledge; however, our ability to analyze and interpret these data lags far behind data generation and storage capacity. Computational models are increasingly used to help interpret biomedical data produced by high-throughput genomics, proteomics, and immunomics projects [1–3]. Advanced applications of computer models that enable the simulation of biological processes are used to generate hypotheses and plan experiments [4–7]. Appropriately interfaced with biomedical databases, computational models enable rapid access to higher-level knowledge and its sharing through data mining and knowledge discovery approaches.

In this special issue, we take an interest in the investigation of the physiology and pathology of the immune responses, particularly the cellular and molecular processes. 

The paper contributed by R. Carvalho et al. presents an approach in which a computational model represents the interaction of mycobacterium infection with the innate immune system in zebrafish. They use the Petri Net formalism to model interaction between key host elements involved in granuloma formation and infection dissemination, defining a qualitative model for the understanding and description of causal relations within this dynamic process. Their systems in biology framework incorporates mathematical modeling to generate and test hypotheses, to perform virtual experiments, and to make experimentally verifiable predictions. This work demonstrates the use of mathematical models that support the study of mechanisms of tuberculosis infection. 

The immune system is able to respond more vigorously to the secondary contact with a given antigen than to the priming contact. Vaccination protocols generally include at least two doses, in order to obtain high antibody titers. In particular, studies performed in transgenic mouse models of Alzheimer's disease have demonstrated that antibodies against beta-amyloid are able to reduce plaques and improve cognition. In mouse models as well as in clinical trials in Alzheimer's disease patients, induction of high titers of anti-beta-amyloid antibodies correlates with the therapeutic efficacy of vaccination. F. Castiglione et al. have analyzed relations between the time elapsed from the first dose (priming) and the second dose (boost) on the antibody titers, coupling *in vivo* experiments with computer simulations to asses the effect of delaying the second injection. 

A major challenge in immunology is the translation of data into knowledge given the inherent complexity and dynamics of human physiology. The physiology and engineering communities have rich histories in applying computational approaches to translate data obtained from complex systems into knowledge of system behavior. J. Klinke II and Q. Wang review how two related engineering concepts, specifically prototyping and “fitness for use,” can be applied to overcome the pressing challenge in translating data into higher-level knowledge of basic immunology for use in practical applications, such as improvement of therapies. These concepts are illustrated using two immunology-related examples: behavior of beta cell mass at the onset of type 1 diabetes and the dynamics of dendritic cells in the lung. 

M. Pennisi presents a mathematical model developed to reproduce the immune response entitled with the combined administration of activated OT1 cytotoxic T lymphocytes (CTLs) and AntiCD137 monoclonal antibodies. This treatment is directed against melanoma in B16 OVA mouse models exposed to a specific immunotherapy strategy. In this paper, two compartments have been modeled: the injection point compartment where the treatment is administered and the skin compartment where melanoma tumor cells proliferate. The outcomes of the mathematical model are in good agreement with the *in vivo* results. In particular the sensitivity analysis highlighted the key role of OT1 CTLs and suggests that a possible reduction of the number of injected antibodies should not affect substantially the treatment efficacy.

Bacterial infections can be acute or chronic. The chronic bacterial infections are characterized by a large bacterial load or by an infection where bacteria grow rapidly. In these cases the immune response is not capable of completely eliminating the infection, leading to the formation of a pattern known as microabscess (or abscess). The microabscess is characterized by an area comprising fluids, bacteria, immune cells (mainly neutrophils), and many types of dead cells. This distinct pattern of formation can only be numerically reproduced and studied by models that capture the spatio-temporal dynamics of the human immune system. B. Pigozzo et al. developed and implemented a computational model to study the process of microabscess formation during bacterial infection.

Cats infected with the feline immunodeficiency virus (FIV) develop an acquired immunodeficiency syndrome (AIDS), similarly to humans infected with HIV. FIV infection causes an acute viremia, which decreases after several weeks, and the appearance of a subpopulation of activated CD8^+^ T cells that we refer to as CD8^*β*low^ cells. The expansion of this activated T cell population is recognized as an important marker of FIV infection and disease. Characterization of the CD8^*β*low^ population's complex pattern of expansion, including its correlation with other disease markers such as viral load, is likely to increase researchers' understanding of FIV infection and AIDS pathogenesis. B. Ribba et al. propose two simple independent mathematical equations to analyze the time evolution of CD8^*β*low^ population size and of viral load during primary infection in cats with FIV. They developed the models using a population approach and mixed-effects regression techniques, based on repeated measurements in more than 100 cats infected with FIV.

Molecular dynamics (MD) simulations have to be sufficiently long to draw reliable conclusions. However, no method exists to prove that a simulation has converged. In the paper contributed by W. Schreiner et al. a method named “lagged RMSD-analysis” is proposed to determine if an MD-simulation has provided a sufficiently precise model. The analysis is based on RMSD values between pairs of configurations separated by variable time intervals *Dt*. 

In summary, these contributions present state of the art modeling of the immune system. The research papers appearing in this special issue will serve as a guide to current developments and a guide to emerging applications in the fascinating field of immune system modeling. 

Ultimately, the utility of computational/mathematical and other quantitative approaches will help provide better healthcare. The seven papers in this volume demonstrate various aspects of modeling of immune processes and preclinical studies of immune responses.

*Francesco Pappalardo* *Vladimir Brusic* *Holger Fröhlich*
